# Review of Haematological Toxicities in Well-Differentiated Neuroendocrine Tumours: A Case Report and Comprehensive Review of the Literature

**DOI:** 10.3390/jcm15124628

**Published:** 2026-06-15

**Authors:** David Gomez, Ramón Salazar, Paula Jiménez Fonseca, Ana Custodio, Beatriz Antón, Amaya Sadaba, Marta Benavent, Ana Elsa Huerta, Barbara Silvia Martinez, Itziar Gomez, Nieves Martínez Lago, Jorge Hernando, Ruth Vera

**Affiliations:** 1 Department of Medical Oncology, Hospital Universitario de Navarra (HUN)-IdiSNA, Calle de Irunlarrea, 3, 31008 Pamplona-Iruña, Spain; amaya.sadaba.diaz@navarra.es (A.S.); ruth.vera.garcia@navarra.es (R.V.); 2 Medical Oncology Department, Oncobell Program IDIBELL Institut Català D’Oncologia (ICO) Duran I Reynals Hospital, Clinical Sciences Department, Bellvitge Campus, University of Barcelona, Centro de Investigación Biomédica en Red de Cáncer, 08007 Barcelona, Spain; ramonsalazar@iconcologia.net; 3 Department of Medical Oncology, Hospital Universitario Central de Asturias, 33011 Oviedo, Spain; palucaji@hotmail.com; 4 Department of Medical Oncology, Hospital Universitario La Paz, 28046 Madrid, Spain; anabcustodio@gmail.com; 5 Department of Medical Oncology, Princess Margaret Cancer Centre, Toronto, ON M5G 2M9, Canada; beatriz.antonp@gmail.com; 6 Department of Medical Oncology, Hospital Universitario Virgen Del Rocío, 41013 Sevilla, Spain; martabenaventv@gmail.com; 7 Translational Medical Oncology Unit, Navarrabiomed-Instituto de Investigación Sanitaria de Navarra (IdiSNA), 31008 Pamplona, Spain; ae.huerta.hernandez@navarra.es; 8 Department of Nurse, Hospital Universitario de Araba, 01009 Vitoria, Spain; barbarasilvia.martinezvalls@osakidetza.eus (B.S.M.); itziar.gomezsanchez@osakidetza.eus (I.G.); 9 Medical Oncology Department, Santiago de Compostela University Clinical Hospital (CHUS) and Health Research, Institute of Santiago de Compostela (IDIS), 15706 Santiago de Compostela, Spain; nievesmartinezlago@gmail.com; 10 Department of Medical Oncology, Hospital Universitario Vall D’Hebron, Vall d’Hebron Institute of Oncology, 08035 Barcelona, Spain; jhernando@vhio.net

**Keywords:** “neuroendocrine tumors” [Mesh], “gastro-enteropancreatic neuroendocrine tumor” [Supplementary Concept], “thrombocytopenia” [Mesh], “adverse effects” [Subheading], “streptozocin” [Mesh], “antineoplastic agents” [Mesh], peptide receptor radionuclide therapy

## Abstract

**Background:** Neuroendocrine tumours (NETs) are heterogeneous neoplasms with several treatment options. Response rates, disease progression, and haematological toxicities can limit the use of some indicated treatments. **Case Presentation:** A 73-year-old woman with a well-differentiated grade 2 pancreatic NET (Ki-67 18%) underwent surgical resection and later developed hepatic recurrence. First-line treatment with sunitinib plus octreotide achieved temporary disease stabilisation. Upon progression, peptide receptor radionuclide therapy (PRRT) with ^177^Lu-DOTATATE was initiated, resulting in stable disease but complicated by grade 3 thrombocytopenia. Two years later, PRRT retreatment was performed due to disease progression, which led to grade 4 thrombocytopenia. Further treatments with capecitabine and everolimus were limited by progression and significant thrombocytopenia. Therapy was switched to streptozocin plus 5-fluorouracil, which resulted in recovery of platelet counts, absence of haematological toxicity, and a sustained radiologic response until March 2025, when she presented with hepatic progression. FOLFOX chemotherapy was initiated but discontinued after one cycle due to severe thrombocytopenia. Deterioration in general condition ultimately led to supportive care and death in March 2026. **Conclusions:** This case highlights the risk of cumulative haematological toxicity with PRRT, particularly in retreatment settings. Careful patient selection and close monitoring are essential. Streptozocin-based chemotherapy may be an effective and well-tolerated alternative for patients with treatment-limiting toxicity.

## 1. Introduction

Neuroendocrine neoplasms (NENs) are a heterogeneous group of tumours arising from neuroendocrine cells of the mucous membranes. The overall incidence of NENs is 2.5/100,000 per year, and 5-year overall survival (OS) is 50% for well-differentiated neuroendocrine tumours (NETs) and 12% for poorly differentiated neuroendocrine carcinomas (NECs) [1]. Approximately 61.0% of NETs are gastro-enteropancreatic (GEP) [2]. Other primary sites for NETs include the lungs, thyroid, ovaries, cervix, pituitary, and adrenal glands [3]. The prevalence of GEP-NENs in Europe ranges from 2.1 to 6.6 cases per 100,000 population [4].

Several effective treatments for NETs exist, each with a different safety profile. Treatment strategies must be determined by the primary tumour, the presence of local and distant metastases, tumour differentiation, associated symptoms or syndromes, and the presence of carcinoid heart disease [5]. Consequently, an interdisciplinary and collaborative clinical approach is necessary to manage NETs.

Surgical intervention is the only potentially curative treatment for localised NETs when size and location make it feasible [6]. For unresectable advanced disease or metastatic disease, multiple therapeutic options are available. The standard first-line treatment for advanced disease is somatostatin analogues (octreotide and lanreotide) [7,8,9]. Peptide receptor radionuclide therapy (PRRT) with 177Lu-DOTATATE is another targeted option for NETs, delivering radionuclides directly to tumour cells [10]. In recent decades, molecular targeted therapies for tumorigenesis, such as the mTOR inhibitor everolimus, the multi-targeted receptor tyrosine kinase inhibitor (TKI) sunitinib, and the vascular endothelial growth factor (VEGF) antibody bevacizumab, have been added to the therapeutic options for progressive NETs [11,12,13].

Chemotherapy with streptozocin- or temozolomide-based regimens is another well-established treatment option. The European Neuroendocrine Tumor Society (ENETS) recommends chemotherapy for unresectable, well-differentiated, progressive, grade 2, symptomatic, or bulky duodenal-pancreatic NETs, as well as for grade 3 NENs [12]. Chemotherapy may also be considered for tumours at other sites (such as the stomach, colon, or rectum) in certain circumstances, including G2 tumours, rapidly progressive disease, treatment failure, or somatostatin receptor-negative tumours.

Despite the availability of this range of therapeutic options for advanced GEP-NETs, response rates remain limited, and many patients experience disease progression and significant haematological toxicities, underscoring the need for new treatment approaches.

Given the limited evidence on haematological toxicity in patients with NETs, despite its frequent occurrence and clinical relevance, we conducted a review of this topic based on a clinical case from our institution thrombocytopenia occurring across nearly all treatment lines administered during the patient’s disease course.

## 2. Detailed Case Description

We present the case of a 73-year-old woman with no significant medical history who, in June 2019, developed abdominal pain. A computed tomography (CT) scan revealed a localised pancreatic mass. The patient underwent cephalic duodenopancreatectomy, and histopathological analysis confirmed a well-differentiated grade 2 pancreatic NET, staged pT3N1M0 according to the TNM classification, with a Ki-67 index of 18%.

**Figure 1:**
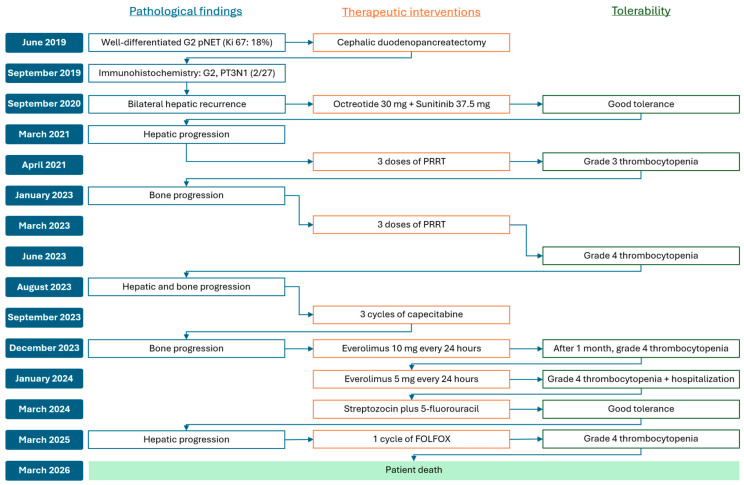
Clinical evolution of the patient.

The course and progression of the disease are summarised in Figure 1. Throughout the case, treatment response was assessed using RECIST 1.1 criteria for CT scans and PERCIST criteria for positron emission tomography (PET) imaging.

FOLFOX, Folinic acid plus 5-fluorouracil plus oxaliplatin; pNET, Pancreatic neuroendocrine tumour; PRRT, Peptide receptor radionuclide therapy.

One year after surgery, radiological follow-up showed bilateral hepatic recurrence, and somatostatin receptor scintigraphy demonstrated hepatic uptake. Consequently, the patient began first-line treatment for advanced disease with 37.5 mg of sunitinib and 30 mg of octreotide every 28 days (cycle 1 of octreotide on 15 October 2020 and cycle 1 of sunitinib on 26 October 2020). She tolerated the treatment well; however, six months after initiation, the disease progressed. In April 2021, she started a new line of treatment with PRRT.

The patient remained radiologically stable, but after three cycles of PRRT (1st dose on 6 June 2021, 2nd on 8 August 2021, and 3rd on 5 October 2021), she developed haematological toxicity, manifesting as grade 3 thrombocytopenia (Table 1).

In January 2023, two years after the last cycle of PRRT, the patient showed progression of bone disease. Fluorodeoxyglucose PET (FDG-PET) showed no evidence of metabolically active tumour disease, whereas Ga-68-DOTATOC PET revealed a Krenning score of 4. Following evaluation by the endocrine tumour board and consideration of both the imaging findings and the previously observed therapeutic benefit, PRRT was reinitiated.

The patient received two additional doses (1st dose on 15 March 2023 and 2nd on 15 June 2023). However, in June 2023, she developed haematological toxicity, presenting with grade 4 thrombocytopenia. The toxicity resolved spontaneously four months after completion of PRRT.

Two months after the last cycle of PRRT, in August 2023, radiological evaluation revealed progression of hepatic and bone disease. Consequently, after thrombocytopenia had resolved in September 2023, third-line treatment with capecitabine (750 mg/m^2^ every 12 h for 14 out of 28 days) was initiated. Temozolomide was not administered because of persistent thrombocytopenia. Treatment was discontinued after three cycles due to further bone progression observed in December 2023.

As a result, fourth-line therapy with everolimus (10 mg every 24 h) was initiated. After one month, the patient had poor haematological tolerance, developing grade 4 thrombocytopenia. Despite a dose reduction to 5 mg every 24 h, hospitalisation was required.

In March 2024, treatment was switched to streptozocin plus 5-fluorouracil (Uppsala regimen), which was well tolerated haematologically, with recovery of platelet counts and no toxicity in other systems. After three months of treatment, a metabolic response was observed in both hepatic and bone lesions.

The patient continued the same treatment regimen, with stable disease until March 2025, when hepatic progression was documented. Given the patient’s preserved general condition (Eastern Cooperative Oncology Group [ECOG] performance status 1), a new line of chemotherapy was initiated with folinic acid, 5-fluorouracil, and oxaliplatin (FOLFOX regimen). However, only one cycle was administered due to poor tolerance, including moderate asthenia and a new episode of thrombocytopenia (platelet count: 19,000/µL).

Due to deterioration in general condition (ECOG performance status 3) and persistent thrombocytopenia, systemic treatment was discontinued, and care was focused on comfort measures and clinical follow-up. After one year without active oncological treatment, the patient died in March 2026 from clinical tumour progression.

During her oncological disease, the patient did not receive any medication known to affect platelet counts, such as anticoagulants or non-steroidal anti-inflammatory drugs. There was no evidence of any concurrent medical condition that could independently exacerbate thrombocytopenia, such as liver disease or hypersplenism. CT and PET imaging showed no evidence of bone marrow infiltration. A bone marrow biopsy was not performed because the patient had isolated thrombocytopenia with normal haemoglobin and neutrophil levels, and platelet counts improved during treatment interruptions. The patient also showed no clinical features of carcinoid syndrome.

## 3. Relevant Sections

### 3.1. Incidence and Nature of Haematological Toxicity

Haematological toxicity is among the most severe, frequent, and well-documented adverse effects of oncological therapy [14,15,16]. It primarily manifests as myelosuppression, including thrombocytopenia, leukopenia, and, ultimately, anaemia. Haematological toxicity is also linked to treatment delays and dose reductions in systemic therapies for oncological diseases, potentially compromising clinical outcomes [17]. Consequently, the risk of haematological toxicity should be carefully evaluated before initiating any therapy.

The incidence and severity of haematological toxicity vary by treatment regimen, study design, and patient population. This publication reviews the clinical trials that led to the approval of therapies for NETs, as well as other studies, and presents relevant data on haematological toxicity (Table 2).

### 3.2. Somatostatin Analogues Haematological Toxicities

Somatostatin analogues, such as octreotide and lanreotide, are used as first-line treatments for grade 1 and grade 2 somatostatin receptor-positive GEP-NETs with a Ki-67 index of up to 10%. Approval of octreotide was based on the results of the PROMID study, in which treatment-naïve patients with well-differentiated metastatic midgut NETs were randomised to receive octreotide or placebo until tumour progression or death. The study demonstrated a significantly longer median time to tumour progression and a higher proportion of patients with stable disease in the octreotide group. Haematological toxicity of any grade was observed in 5 of 42 patients receiving octreotide, although the specific type of toxicity was not detailed [9].

Lanreotide is another established somatostatin analogue for advanced GEP-NETs. Its efficacy was demonstrated in the phase 3 CLARINET study, which showed significant improvements in PFS versus placebo in patients with GEP-NETs and Ki-67 < 10% [7]. The CLARINET FORTE trial evaluated the efficacy and safety of a reduced lanreotide dosing interval (120 mg every 14 days) in patients with progressive midgut or pancreatic NETs after first-line standard-dose (120 mg every 28 days) and found a significant improvement in PFS. The authors reported a 3% discontinuation rate due to toxicity and did not provide more detailed haematological toxicity data [18].

### 3.3. PRRT Haematological Toxicities

The NETTER-1 and NETTER-2 trials are randomised controlled trials evaluating the efficacy and safety of lutetium-177 (177Lu)–Dotatate in patients with advanced, somatostatin receptor–positive midgut NETs [10,19]. NETTER-1 assessed patients who had progressed after first-line somatostatin analogue therapy, while NETTER-2 evaluated treatment in treatment-naïve patients. Both trials demonstrated favourable outcomes with PRRT, with improvements in progression-free survival (PFS) and OS. Clinical benefit has been reported in patients with grade 1–3 GEP NETs and Ki-67 indices up to 55%. NETTER-1 and NETTER-2 reported objective response rates (ORR) of 18% [10] and 43% [19], respectively.

According to the NETTER-1 trial, neutropenia occurred in 5% of patients treated with PRRT, anaemia in 14%, and thrombocytopenia in 25%. Grade 3 or 4 neutropenia and thrombocytopenia were reported in 1% and 2% of PRRT-treated patients, respectively, compared with none in the control group. Grade 3–4 lymphopenia affected 9% of patients receiving PRRT, while no cases of grade 3–4 anaemia were observed [10].

The NETTER-2 study reported an incidence of 2% for neutropenia of any grade, less than 1% for anaemia, and 12% for thrombocytopenia. Grade 3 or higher haematological toxicity occurred in 14% of patients, with anaemia in less than 1%, and neutropenia and thrombocytopenia in 2% [19].

Sabet and colleagues reported higher long-term incidences of grade 3–4 haematological toxicity in a large cohort of patients undergoing PRRT for metastatic NETs. The incidence of grade 3–4 thrombocytopenia, leucopenia, and anaemia was 4.9%, 6.4%, and 3.4%, respectively [20].

Other studies have documented a broader range of haematological toxicities, with leukopenia and anaemia affecting up to 25% and 10% of patients, respectively [21]. The Spanish SEPTRALU (Spanish Series of Patients Treated with Lutetium Radionuclide-177) registry, which collects real-world data on patients treated with PRRT, reported that 29.8% experienced any-grade haematological toxicity and 4.7% had grade 3 or higher toxicity. These figures are notably higher than those reported in the NETTER-1 and NETTER-2 studies [21].

The risk of myelodysplastic syndrome (MDS) or acute leukaemia, as reported by Sabet et al. and Botei et al., was 2% [20,22]. In the NETTER-1 trial, one patient (0.9%) in the treatment group developed MDS, possibly related to the investigational therapy. Consequently, although the risk of MDS or acute leukaemia is low, long-term monitoring of haematological parameters is essential for patients receiving PRRT.

### 3.4. Chemotherapy Haematological Toxicities

The combination of fluoropyrimidines (5-fluorouracil or capecitabine) with streptozocin or temozolomide is a systemic treatment option for GEP-NETs. Streptozocin and 5-fluorouracil are indicated for grade 1 and grade 2 pancreatic NETs. Although capecitabine and temozolomide (CAPTEM) are not officially approved for NETs, they are commonly used off-label for pancreatic NETs of any grade and for intestinal NETs with a Ki-67 index < 10%.

Haematological toxicity is less common with streptozocin than with other chemotherapy agents. However, this risk increases when streptozocin is combined with other drugs, such as 5-fluorouracil. The haematological toxicity of the streptozocin–5-fluorouracil combination has been evaluated in two randomised clinical trials [23,24]. Haematological toxicity was more frequent in the combination arm than in the control arm: leukopenia was reported in 25–73% of patients, and thrombocytopenia in 27%.

The SEQTOR study evaluated the combination of streptozocin and 5-fluorouracil against everolimus in patients with grade 1 or 2 pancreatic NETs. The study demonstrated the benefit of the combination, with an ORR of 30%. ORR was significantly higher in subgroups with ECOG performance status 0, grade 2 tumours, Ki-67 > 2%, and prior treatment. Grade 3 or higher haematological toxicity in the streptozocin and 5-fluorouracil group was as follows: 7.5% for neutropenia and 0% for thrombocytopenia. Neutropenia of any grade occurred in 9.7% of patients, thrombocytopenia in 10.6%, and anaemia in 4.4% [25].

The E2211 study compared temozolomide with capecitabine plus temozolomide in patients with advanced low- or intermediate-grade pancreatic NETs. The combination significantly improved PFS compared with temozolomide alone. The discontinuation rate due to any toxicity was higher in the capecitabine plus temozolomide arm (14.9%) than in the control arm (6.3%). Across the study, 37% of patients experienced grade 1–2 anaemia, and 1% had grade 3–4 anaemia. Grade 1–2 thrombocytopenia was observed in 28% of patients, with grade 3–4 thrombocytopenia occurring in 10%. Additionally, 15% of patients had grade 1–2 neutropenia, and 18% had grade 3–4 neutropenia [26].

#### 3.4.1. Everolimus Haematological Toxicities

The RADIANT-2 trial evaluated the efficacy and safety of everolimus combined with octreotide LAR versus octreotide LAR alone in patients with advanced NETs associated with carcinoid syndrome [27]. The everolimus group showed a median PFS improvement of 5.1 months compared with placebo, although no significant difference in OS was observed [27,28]. In the everolimus arm, the incidence of any-grade anaemia and thrombocytopenia was 16.3% and 14.9%, respectively, while grade 3–4 toxicity affected 1.4% and 4.2% of patients [27].

Subsequently, the RADIANT-3 trial compared everolimus with placebo in patients with advanced, progressive, low- or intermediate-grade pancreatic NETs. Everolimus improved median PFS by 6.4 months and was associated with a statistically nonsignificant survival benefit of 6.3 months [29,30]. Haematological toxicity was comparable to that reported in RADIANT-2, with grade 3–4 anaemia and thrombocytopenia occurring in 4.9% and 3.9% of patients, respectively. Although neutropenia was reported as one of the most common grade 3–4 drug-related adverse events, the exact percentage of patients experiencing it was not provided [30]. Similarly, the RADIANT-4 trial, which included patients with advanced, progressive, well-differentiated, non-functional NETs of lung or gastrointestinal origin, reported anaemia in 16% of patients, with grade 3–4 anaemia occurring in 4% [31].

#### 3.4.2. Sunitinib Haematological Toxicities

The effect of sunitinib in the treatment of pathologically confirmed, well-differentiated, advanced or metastatic pancreatic NETs in patients ineligible for surgery was evaluated in the SUN-1111 study. Sunitinib demonstrated improved PFS, OS, and ORR compared with placebo. Any-grade neutropenia was observed in 29% of patients in the sunitinib group, with grade 3–4 neutropenia affecting 12%. The incidence of any-grade thrombocytopenia was 17%, and grade 3–4 thrombocytopenia occurred in 4% of patients in the treatment arm. The primary toxicity associated with sunitinib was gastrointestinal [32]. Currently, sunitinib is approved only for patients with pancreatic NETs.

### 3.5. Cabozantinib Haematological Toxicities

The CABINET study evaluated the efficacy and safety of cabozantinib in two cohorts: patients with extra-pancreatic NETs and those with metastatic pancreatic NETs after progression on one or more prior lines. Cabozantinib improved PFS in both cohorts, with the greatest benefit observed in patients with grade 2 extra-pancreatic NETs and grade 2–3 pancreatic NETs. In the pancreatic NETs cohort, thrombocytopenia of any grade was reported in 33% of patients, with no grade 3–4 events observed. Neutropenia of any grade occurred in 27% of patients, including 2% with grade 3–4. Anaemia was not reported. In the extra-pancreatic NET cohort, thrombocytopenia was more common (47% of any grade and 1% grade 3–4), whereas the neutropenia rate was compared to the pancreatic cohort. Anaemia was reported in 21% of patients of any grade, including 2% grade 3–4 [33].

## 4. Discussion

This case report describes a patient with an advanced grade 2 pancreatic NET who progressed after first-line treatment with sunitinib and octreotide. After disease progression, the patient received PRRT, which was effective but caused grade 3 thrombocytopenia. Two years later, the patient underwent retreatment with PRRT, resulting in more severe thrombocytopenia.

According to the studies that led to the approval of the different drugs, neutropenia is more frequently associated with TKIs such as sunitinib or cabozantinib; thrombocytopenia is more common with PRRT and CAPTEM; whereas anaemia is more commonly observed with CAPTEM or everolimus.

PRRT is an established treatment for advanced NETs that overexpress somatostatin receptors, given its positive impact on PFS and OS. However, careful patient selection is crucial when considering PRRT versus alternative therapies, such as chemotherapy. Identifying the most suitable patient profiles is essential. In cases of high tumour burden, symptomatic disease, or greater FDG-PET uptake than Ga-68 DOTATOC PET, chemotherapy may be preferred due to its faster mechanism of action and quicker tumour response [34,35].

Several studies have reported that additional PRRT cycles in the so-called salvage therapy setting are feasible, safe and effective [36,37,38,39]. Consequently, beyond selecting patients for initial PRRT, another critical aspect is determining eligibility for retreatment. One key factor is the duration of clinical benefit: some studies require a PFS of at least 12 months after the last PRRT cycle [40,41,42], whereas others suggest a minimum of 18 months [36].

Another key challenge of PRRT compared with other treatments is its potential for long-term toxicity. While adverse effects from agents such as everolimus, sunitinib, or chemotherapy typically arise during treatment and resolve after discontinuation, PRRT-related toxicities can be unpredictable, sometimes emerging months later and complicating long-term management. Previous studies indicate that acute side effects of PRRT are generally mild and self-limiting, with nausea the most common. In contrast, long-term toxicities may include renal dysfunction, myelodysplastic syndrome, and acute leucopenia, with haematological toxicity considered the most significant adverse event following PRRT. Consequently, follow-up after PRRT can be more complex than with other therapies, as treatment responses tend to be delayed, making it challenging to establish an optimal follow-up strategy [22,43].

In this case, dose reduction was used to mitigate toxicity during third-line everolimus treatment. After the patient developed grade 4 thrombocytopenia, the everolimus dose was reduced from 10 mg to 5 mg daily. However, in some cases, dose adjustments alone are insufficient to manage myelosuppression, necessitating a change in treatment. This was the case for our patient, who transitioned to chemotherapy with streptozocin and 5-fluorouracil after everolimus.

The route of administration is another factor to consider when selecting a therapy for NETs. Studies suggest that treatment preferences vary by educational level. A common misconception is that oral therapies are less effective and have fewer adverse effects than intravenous treatments. Interestingly, patients with higher levels of education (e.g., a graduate degree or equivalent) tend to prefer oral therapies. However, research indicates that toxicity levels are similar regardless of the route of administration, challenging this belief [44,45]. It has also been observed that women may be at higher risk of myelotoxicity when treated with CAPTEM, particularly after prior PRRT. In these patients, higher rates of grade 4 thrombocytopenia and neutropenia have been reported compared with men [46].

Finally, although much of the haematological toxicity data for NET treatment comes from clinical trials, real-world practice often shows higher toxicity rates, as seen in comparisons between SEPTRALU and NETTER-1 and NETTER-2. Clinical trials typically enrol patients with better overall health, fewer comorbidities and less polypharmacy than those treated in everyday medical settings. As a result, haematological toxicity may be more frequent in real-world practice than reported in randomised clinical trials.

## 5. Conclusions

In conclusion, multiple oncological treatments for NETs have demonstrated efficacy, significantly improving survival rates. Therefore, close monitoring of adverse effects is essential for selecting the most appropriate therapeutic option for each patient, ultimately enhancing quality of life and minimising treatment discontinuation.

## Figures and Tables

**Table 1:** Platelet counts throughout the clinical case.

Date	Therapeutic Situation	Platelet Count (Platelets/µL)
April 2021	Before the 1st cycle of PRRT	118.000
November 2021	After the 3rd cycle of PRRT	37.000
January 2022	Recovery period after PRRT	56.000
August 2022	Recovery period after PRRT	102.000
February 2023	Before the 1st cycle of retreatment with PRRT	108.00
July 2023	After the 2nd cycle of retreatment with PRRT	23.000
September 2023	Before the 1st cycle of capecitabine (no temozolomide due to thrombocytopenia)	50.000
December 2023	Before the 1st cycle of everolimus 10 mg	44.000
January 2024	After the 1st cycle of everolimus 10 mg	15.000
February 2024	Recovery period after everolimus 10 mg	49.000
	After the 1st cycle of everolimus 5 mg	16.000
March 2024	Recovery before the 1st cycle of STZ-5FU	52.000
March 2025	Before the 1st cycle of FOLFOX	75.000
April 2025	Before treatment with FOLFOX	19.000

FOLFOX, Folinic acid plus 5-fluorouracil plus oxaliplatin; PRRT, Peptide receptor radionuclide therapy; STZ-5FU, Regimen of streptozocin and 5-fluorouracil combined.

**Table 2:** Incidence of haematological toxicity of NETs therapeutic options reported in pivotal studies.

		Anaemia	Thrombocytopenia	Neutropenia
PRRT	All grades	1–14%	12–25%	2–5%
	Grades 3–4	0–1%	2%	2%
Everolimus	All grades	16.3%	14.9%	NP
	Grades 3–4	1.4–4.9%	3.9–4.2%	NP
Sunitinib	All grades	18%	17%	29%
	Grades 3–4	4%	4%	12%
Cabozantinib	All grades	Panc: NP ExtraPanc: 21%	Panc: 33% ExtraPanc: 47%	Panc: 27% ExtraPanc: 30%
	Grades 3–4	Panc: NP ExtraPanc: 2%	Panc: 0% ExtraPanc: 1%	Panc: 2% ExtraPanc: 3%
STZ-5FU	All grades	4.4%	10.6%	9.7%
	Grades 3–4	0%	0%	7.5%
CAPTEM	Grades 1–2	37%	28%	15%
	Grades 3–4	1%	10%	18%

CAPTEM, Regimen of capecitabine and temozolomide combined; ExtraPanc, Extra-pancreatic NET Cohort; NP, Not provided; Panc, Pancreatic NET Cohort; PRRT, Peptide receptor radionuclide therapy; STZ-5FU, Regimen of streptozocin and 5-fluorouracil combined.

## Data Availability

The data presented in this study are available on request from the corresponding author.
